# Medical Education and the University of London

**Published:** 1904-03-12

**Authors:** 


### Medical Education and the University of London.

The efficiency of medical education in London is no mere sectional or local interest. It concerns indeed many influences which touch the general welfare. Inevitably it must be the case that a considerable number of students will continue to obtain their professional training at metropolitan schools, hence it is a matter of national concern that suitable and adequate opportunities be provided for these students. A limited and imperfect medical education means not only lack of progress in the medical art but also inefficient individual practitioners. Now it will be generally recognised that in practical hospital work London presents a field of opportunity which has no parallel in this country. Its clinical services, too, are organised by physicians and surgeons many of whom are in the front rank of professional eminence as regards learning, ability, and teaching capacity. It need not be claimed that the organisation of the schools for clinical instruction is perfect, but it is beyond question that in all the practical departments the student is offered every chance to make himself acquainted with the phenomena and treatment of disease and thoroughly to equip himself for the practice of his profession. Another and very different picture is, however, presented when attention is directed to the equipment of the schools in regard to the teaching of the earlier subjects of the medical curriculum. Here it is admitted the educational apparatus is seriously defective. The defect, too, is inherent in the nature of the existing organisation. Each of the schools, if it would hold its position secure beside its rivals, is impelled to offer instruction in all the several branches of the medical curriculum. Consequently it must provide teachers in such subjects as physics, chemistry, anatomy, and physiology. But in most instances there are absolutely no funds for this purpose. It is inevitable in such circumstances that the teaching of these subjects must suffer. That in spite of much discouragement and many difficulties individual teachers have done and continue to do good work may be allowed. But the very nature of the case is opposed to the development of a high and sustained level, and no one acquainted with the facts will argue that this, under the existing system, either has been or can be generally attained. The position of recent years has become more acute because of the enormous development of the sciences on which medicine and surgery as practical arts rest and because also of the closer association of these arts with scientific knowledge. It is nowadays impossible for anyone engaged in professional work as a physician or surgeon to be at the same time an efficient teacher of physical, chemical, or biological science. Each one of these subjects demands an undivided energy. Yet hardly any one of the metropolitan schools is in a position to offer to teachers of these subjects more than a scanty pittance, and none certainly can compete in this respect with the Scottish and certain of the provincial schools. The position therefore is that London at the present moment possesses some twelve medical schools, no one of which can boast a satisfactory equipment for the teaching of the earlier and intermediate, but none the less essential, branches of medical science. To the removal of this unfortunate state of matters the authorities of the University of London have determined to address themselves. As was to be expected, they realise that upon the adequate development of efficient scientific teaching the future of the metropolis as a great centre of medical education depends. This development cannot be expected in the existing schools. Absence of funds, if no other reason, blocks the way. Further, there is the less need that any individual school should make the attempt, seeing that the scientific subjects of the curriculum can be as readily taught to large as to small classes of students. Hence centralisation is demanded by considerations of economy not less than by the desire for efficiency. All this in principle is conveyed in the proposal to establish and endow an Institute of Medical Sciences under the control of the University of London. That such an institution, if adequately endowed, will be able to secure the most distinguished and capable teachers is certain. Equally it secures that under the direction of the University the teaching would be carried along sound lines, and would include the most modern laboratory and practical opportunities. For the existing schools there will remain the splendid field of clinical training which they will be able to cultivate all the more successfully when relieved from responsibilities beyond their capacity and resource. It will indeed be strange if the appeal for support recently issued by the University of London on behalf of their scheme should fail for lack of funds. To those concerned with the large interests of humanity as well as to those anxious for the advance of medicine and science the opportunity to place medical education in London in a worthy and efficient position must surely commend itself.

